# Exploring Chinese EFL Learners' Achievement Emotions and Their Antecedents in an Online English Learning Environment

**DOI:** 10.3389/fpsyg.2021.722622

**Published:** 2021-10-15

**Authors:** Yingli Yang, Zihan Gao, Yawen Han

**Affiliations:** ^1^School of International Studies, University of International Business and Economics, Beijing, China; ^2^School of Foreign Languages, Southeast University, Nanjing, China

**Keywords:** achievement emotions, control-value theory, antecedents, online English learning environment, Chinese EFL learners

## Abstract

Drawing on the control-value theory, this study adopted a qualitative approach to explore the various achievement emotions Chinese EFL learners experienced in an online English learning environment and their antecedents during the COVID-19 pandemic period. Data were collected from six Chinese EFL students through semi-structured interviews and reflective journals supplemented with their class notes. Thematic analysis was performed using the qualitative data management software NVivo 12 plus. Results showed that the students experienced diverse emotions such as enjoyment, relaxation, anxiety, guilt, boredom and helplessness. Apart from the environmental antecedents of teacher and peer factors and individual antecedents of control-value appraisals, four novel antecedents were identified which had influence on emotions experienced in the online learning context, including environmental antecedents of internet connection and workload outside classroom, as well as the individual antecedents of students' self-regulation of learning behavior and learning environment.

## Introduction

The past three decades have witnessed an increasing number of studies focusing on emotions in second language (L2) teaching and learning (Dewaele and MacIntyre, 2014; Li et al., 2019; Shao et al., 2020a). Apart from L2 anxiety which has been extensively studied since Horwitz et al. (1986), researchers have also begun to present a holistic view of L2 emotions with the development of positive psychology (Li et al., 2019). Various emotions in L2 classroom have been explored in an attempt to find out the relationship between L2 emotions and L2 achievement, including positive emotions such as enjoyment, love, hope, interest, as well as negative emotions such as boredom, shame and guilt (Teimouri, 2018; Li et al., 2019). For example, Li et al. (2019) found that enjoyment had a significant positive correlation with English language performance, while anxiety was negatively correlated with English language performance in both the middle and high achievement groups. Teimouri (2018) reported that shame was negatively correlated with L2 motivation and achievements, while guilt had positive effects on language achievements. Emotions have also been found to have the impact on L2 learning and performance by influencing students' attentional processes such as memory and information processing (Lantolf and Swain, 2019), enhancing or decreasing students' interest and motivation (Li et al., 2019), facilitating or impeding students' engagement (Oga-Baldwin, 2019) and affecting self-regulated learning (Artino and Jones, 2012).

Considering the important role of emotions in L2 learning, researchers have also conducted studies on their antecedents (i.e., the reasons) to find appropriate methods to facilitate positive L2 emotions and reduce negative ones (Dewaele et al., 2017; Chen, 2020; Li, 2021). Most of the studies investigating the antecedents of L2 learner emotions focused on emotions such as enjoyment, anxiety and boredom (Dewaele et al., 2017; Jiang and Dewaele, 2019, 2020; Li, 2021). For example, Dewaele et al. (2017) investigated learner and teacher variables for L2 enjoyment and anxiety. Li (2021) explored the role of perceived control and value in predicting L2 boredom. However, according to Oxford (2016), language learners unfolded complex emotional experiences which can not be simplified into just one or two emotions. Therefore, it is necessary to investigate the antecedents of a wide variety of emotions that occur in the L2 learning process.

In addition, although there has been a great number of studies on L2 emotions conducted in the traditional face-to-face classroom, studies on emotions in the online language teaching and learning context are still limited (Russell, 2020). With the breakout of COVID-19, schools worldwide shifted to online teaching and learning abruptly, solving the problem of physical distance and guaranteeing the teaching schedule. Online learning emotion was one of the key factors that influenced the effect of online teaching since emotions play a critical role in L2 achievement (Dewaele et al., 2019a). Online class is different from offline class in terms of learning environment, learning medium, classroom interaction and perceived social support (Alonso Díaz and Blázquez Entonado, 2009; Artino and Jones, 2012; Coman et al., 2020; Russell, 2020), which may lead to different emotions. More specifically, students in the online learning environment may encounter physical isolation in space and emotional isolation from peers and teachers, which results in less desirable learning outcome (Russell and Murphy-Judy, 2020).

In order to extend our understanding of L2 achievement emotions in the new context of online learning, we examined various achievement emotions experienced by six EFL learners in mainland China during the COVID-19 pandemic when all the students took online English classes for the whole semester. Furthermore, we explored the antecedents of these different emotions with the framework adapted from Pekrun and Perry (2014).

## Literature Review

### Achievement Emotions

Achievement emotions refer to the various emotions experienced in the academic settings ubiquitously which are “tied directly to achievement activities or achievement outcomes” (Pekrun, 2006, p. 317). According to Pekrun (2006), achievement emotions can be classified into a three-dimensional taxonomy, including object focus, valence and arousal. This framework has been applied extensively to distinguish different achievement emotions by researchers from various research contexts (Putwain et al., 2018; Shao et al., 2020b; Li, 2021). Of the three dimensions, the two dimensions of valence and arousal are perceived to be the most important in describing emotions and the performance effect (Pekrun et al., 2007). Positive or negative emotions are related to the valence of achievement emotions. And the activating or deactivating emotions pertain to the different levels of arousal. Using the two dimensions, four basic categories of emotions can be formed, including positive activating emotions, positive deactivating emotions, negative activating emotions and negative deactivating emotions. For example, hope belongs to positive activating emotions while hopelessness belongs to negative deactivating emotions. The two-dimensional taxonomy of achievement emotions is presented in Table 1.

**Table 1:** A two-dimensional taxonomy of achievement emotions (adapted from Pekrun et al., 2007).

	**Positive**	**Negative**
Activating	Hope	Anxiety
	Enjoyment	Guilt
Deactivating	Relaxation	Boredom
	Contentment	Hopelessness

### Control-Value Theory (CVT) of Achievement Emotions

Considering the significant role of the achievement emotions, Pekrun (2006) put forward the CVT to further investigate the antecedents of achievement emotions, the outcome of these emotions, and ways to regulate emotions. The present study will mainly focus on the antecedents of achievement emotions, covering two corollaries of the theory. Firstly, the CVT holds that individual control-value appraisals are at a central place to arouse the achievement emotions. Secondly, there exist other individual and environmental antecedents which can influence the resulting emotions.

Control-value appraisals are of specific relevance to achievement emotions (Pekrun, 2006). Control appraisals refer to the perceived controllability over actions and outcomes while value appraisals refer to the subjective importance of the academic activities and outcomes. Value appraisals include appraisals of intrinsic value which relates to perceiving the value *per se* and extrinsic value which emphasizes the instrumental utility of academic activities or outcomes.

Achievement emotions can be caused by control-value appraisals and different control-value appraisals can instigate different emotions. According to Pekrun and Perry (2014), emotions can be triggered by different levels of perceived control, perceived value or their interaction. For example, hopelessness is experienced when there is a complete lack of perceived control of the activity, anger appears when there is perceived negative intrinsic or extrinsic value of the activity, and enjoyment is assumed to depend on an interaction of positive control and value appraisals of the activity and learning outcomes. Many researchers explored the antecedents of different achievement emotions based on the control-value appraisals (Camacho-Morles et al., 2019; Ghensi et al., 2021). Putwain et al. (2018) explored the control-value appraisals as the antecedents of enjoyment and boredom and found that control and value appraisals can positively predict enjoyment while negatively predict boredom. In addition, the interaction of control and value appraisals can lead to different emotions. For example, perceived high control was generally related to enjoyment; however, if the student perceived high control and low value, he might experience boredom.

Other individual antecedents such as temperament and gender as well as environmental antecedents can also arouse achievement emotions (Pekrun, 2006). The environmental antecedents mentioned in the CVT cover a broad variety of teacher and peer factors such as cognitive quality, motivational quality, autonomy support, and feedback, which can influence emotions by shaping control-value appraisals and then lead to different emotions (Pekrun and Perry, 2014).

### Achievement Emotions in Second Language Acquisition

Since the seminal work on anxiety by Horwitz et al. (1986), researchers have studied a broader variety of L2 emotions covering positive activating emotions, positive deactivating emotions, negative activating emotions and negative deactivating emotions (Pavelescu and Petrić, 2018; Teimouri, 2018; Han and Hyland, 2019; Li and Xu, 2019; Jiang, 2020; Li, 2021). And the relationship between these emotions and L2 performance drew researchers' attention (Li et al., 2019; Resnik and Dewaele, 2020; Shao et al., 2020b).

Empirical data from previous studies generally show that positive activating emotions such as enjoyment and hope can positively affect L2 learning and performance, while negative deactivating emotions such as boredom and hopelessness have a negative effect on L2 learning and performance (Dewaele and Alfawzan, 2018; Li, 2021).

However, controversies exist regarding the role of negative activating emotions such as anxiety, guilt and shame (Marcos-Llinás and Garau, 2009; Teimouri, 2018; Li et al., 2019). As for anxiety, researchers reported contradictory results in terms of whether anxiety exerts a debilitating effect (Oya et al., 2004; Shao et al., 2013; Li et al., 2019), a facilitating effect (Marcos-Llinás and Garau, 2009; Trebits, 2016), or no effect on L2 achievement (In'nami, 2006). Zhang's (2019) meta-analysis showed that anxiety was overall negatively correlated with L2 performance and that the controversies among previous studies may be explained by the moderator variables such as the anxiety type, age, and lexical similarity. As for guilt and shame, Teimouri (2018) revealed that shame was negatively correlated with L2 achievements by lowering students' willingness to communicate, whereas guilt positively affected L2 achievements by motivating students' corrective actions.

Apart from the afore-mentioned three types of emotions (positive activating emotions, negative activating emotions and negative deactivating emotions) which have been explored to a large extent, studies on positive deactivating emotions such as relaxation, calmness and contentment remain limited (Linnenbrink-Garcia and Barger, 2014). One exception was Han and Hyland (2019) which investigated the academic emotions in the situation of written corrective feedback and revealed that students could experience relief and tranquility when they responded to the teacher's feedback. As students may experience various kinds of emotions in the classroom and online learning context, it is necessary to examine these emotions in a comprehensive way to reflect the complexity of students' emotional experiences and various antecedents (Rich, 2016).

A number of studies have explored the antecedents of L2 achievement emotions based on existing or adapted questionnaires (Dewaele et al., 2017; Jin and Dewaele, 2018; Jiang and Dewaele, 2019, 2020; Pawlak et al., 2020). For example, in terms of positive activating emotions, Dewaele et al. (2017) and Jiang and Dewaele (2019) showed that learner internal variables (e.g., gender, L2 proficiency) and teacher related variables (e.g., frequency of the teacher's use of L2, teacher predictability) exert an effect on L2 enjoyment. Regarding negative activating emotions, previous studies have shown that learner internal variables, perceived social support (i.e., emotional support from teacher and peers), and socio-biographical variables (e.g., experience abroad) can lead to L2 anxiety (Dewaele et al., 2017; Jin and Dewaele, 2018; Jiang and Dewaele, 2020). As for negative deactivating emotions, Pawlak et al. (2020) extracted two factors underlying boredom: “disengagement, monotony and repetitiveness” and “lack of satisfaction and challenge”. However, to a large extent, the studies on the causes of emotions are not theorized very well (Dewaele and Li, 2020).

More recently, L2 researchers have adopted the CVT to analyze the antecedents. Shao et al. (2020b) examined the relationship between control-value appraisals, achievement emotions and L2 performance. The results revealed that perceived control and value were both positively related to positive emotions and negatively to negative emotions. Furthermore, control and value appraisals can be interacted to predict achievement emotions. Li (2021) adopted a control-value theory approach to investigate the antecedents of L2 boredom. She found that the appraisals of both control and value were negatively related to boredom in general, and the appraisals of intrinsic value played a larger role than that of extrinsic value and control in predicting boredom. However, these studies only focus on the individual antecedents of control-value appraisals while ignoring the other individual and environmental antecedents.

### Online Language Learning and L2 Emotions

In the wake of COVID-19 pandemic, language learning has been carried out in the online platform for about a semester in many countries. Compared with face-to-face learning, online learning bears the following distinguished features. First, online learning depends greatly on technical conditions. According to Coman et al. (2020), students reported a number of technical problems, including poor internet connection, delayed message viewing, blurred sound and signal loss, all of which can reduce the effect of online teaching. Second, there were fewer interactive activities with teachers and peers such as think-pair-share in the online learning platforms (Russell, 2020). In addition, in the online learning environment, students may experience both physical isolation (e.g., space and time) and psychological emotional isolation from peers and teachers, which requires more motivation and self-disciplinary ability (Russell and Murphy-Judy, 2020). Emotional isolation can be aroused when students perceive less emotional support which refers to perceived likes and cares from peers and teachers (Johnson and Johnson, 1983). Unlike traditional face-to-face classroom where teachers can show their emotional support through facial expressions and body language such as smiling and patting on students' shoulder (Daniels and Stupnisky, 2012; Jiang, 2020), online language learning especially through the instructional mode of audio conferences may lead to less emotional support and resonance (Resnik and Dewaele, 2021). Therefore, students in the online learning context may experience different emotions or different degrees of emotions compared with their counterparts in the face-to-face learning environment even with the same motivation and self-disciplinary ability. While there is a growing interest in investigating L2 students' achievement emotions in traditional face-to-face classrooms, their emotional experiences in the process of online English learning have been largely overlooked.

The few studies focusing on online L2 emotions mostly concern L2 anxiety and enjoyment (Pichette, 2009; Martin and Alvarez Valdivia, 2017; Resnik and Dewaele, 2021). Pichette (2009) compared the anxiety level between first-semester students and more experienced students in the traditional face-to-face classroom and online L2 learning environment. It was found that the level of L2 anxiety was similar in the traditional learning environment for both groups of students while the more experienced distance learners had lower anxiety than their first-semester counterparts. Martin and Alvarez Valdivia (2017) specifically focused on the online oral synchronous communication task. They investigated the relationship between corrective feedback and foreign language anxiety and showed that the high-anxiety group and low-anxiety group preferred different sources of feedback. Resnik and Dewaele (2021) explored L2 enjoyment and anxiety in the “in-person” English teaching classes and emergency remote teaching classes. Results showed that L2 enjoyment and anxiety were both positively correlated in the two settings and that students reported lower level of enjoyment and anxiety in emergency remote teaching classes.

Previous studies on L2 achievement emotions generally demonstrate that positive activating emotions can enhance L2 performance, negative deactivating emotions do harm to L2 performance, and negative activating emotions can play either a facilitating or debilitating role. However, the antecedents of emotions explored from a theoretical perspective receives scant attention. Besides, researchers have found that online learning is different from face-to-face learning in various ways (e.g., learning space, interaction). Therefore, it is necessary to explore the achievement emotions and their antecedents in an online learning environment. To address these research gaps, our study aimed to answer the following two research questions.

RQ1: What achievement emotions do students experience in an online language learning environment?
RQ2: What individual and environmental antecedents account for these emotions experienced in the online language learning environment?

## Methodology

### Research Context

The study was conducted in mainland China where attending English-related courses was compulsory for all the university students (Tao and Gao, 2018). Students pursuing a bachelor's degree are required to take courses such as college English, integrated English course or English listening and speaking to help improve their listening, speaking, reading and writing skills. They also need to pass College English Test Band 4 and Band 6 (CET 4 and CET 6) as measurement for their English proficiency. As for master and doctoral students, academic English courses (e.g., academic English writing, English for international academic communication) are necessary since these students are usually required to publish high-quality papers in international journals and make presentations in English at international conferences.

The English courses were usually carried out in the traditional face-to-face classrooms. However, because of the breakout of COVID-19 pandemic which occurred at the beginning of 2020, the Ministry of Education of China issued the notice calling for online teaching for students at all educational levels in the spring semester of 2020. The English courses were shifted to the online platform in the lockdown situation and lasted for one whole semester from February to July, 2020. Some commonly used synchronous online platforms included Tencent Meeting, Zoom, DingTalk and WeChat. Teachers can choose the instructional mode of video conferences, audio conferences, or teach by sending texts.

### Research Design

The study adopted a qualitative approach since emotions are contextual and should be viewed together with the experiences learners are engaged in, and it is appropriate to dig into the experiences and emotions by using qualitative methods such as interviews (Rich, 2016; Rawal and De Costa, 2019). To get a holistic view and richer information on the achievement emotions Chinese EFL learners experienced and their antecedents, participants were selected by maximum variation sampling and convenience sampling (Dörnyei, 2007). Maximum variation sampling can allow the researchers to explore not only diverse variations but also important common patterns between participants (Patton, 1990). Dörnyei (2007) further assumed that the common pattern across the sampled diversity showed the pattern's reasonable stability. Various kinds of data were collected, including semi-structured interviews, reflective journals and class notes. Thematic analysis was then carried out based on Braun and Clarke's (2006) six-phase-approach. The data were analyzed based on a framework adapted from Pekrun and Perry (2014) to fit the online context.

### Participants

Participants were selected according to the following criteria. Firstly, they were from different educational backgrounds based on the principle of maximum variation sampling (Dörnyei, 2007). More specifically, they were from universities of different types, ranging from highly-ranked “Double First-Class” universities^1^ to non- “Double First-Class” universities. Meanwhile, they majored differently in science and engineering as well as social sciences. In addition, they were pursuing different degrees including graduation certificate, bachelor's degree, master's degree and doctor's degree. Secondly, they took part in an online English class for one whole semester from February to July in 2020. Thirdly, females and males were both selected since gender is a factor that may lead to different achievement emotions (Dewaele et al., 2016). With the help of the researcher's friends who taught English in different universities, we finally got access to six students meeting the above requirements. The information of the participants is detailed in Table 2.

**Table 2:** Description of participants.

**P**	**Gender**	**Age**	**SREP**	**University level**	**Degree pursuing**	**Major**	**English course**	**Instruction medium**
A	Female	20	L	DFC	Bachelor	Media	CE	Chinese/ English
B	Female	25	A	Non DFC	Master	Education	AEW	English
C	Male	27	L	DFC	Doctor	Engineering	AEW	English
D	Male	26	M	DFC	Doctor	Law	EIAC	Chinese/ English
E	Female	19	L	Non DFC	Graduation Certificate	Finance	E	Chinese/ English
F	Female	22	M	DFC	Master	Materials	ELS	English

*P, Participant; SREP, Self-rated English proficiency; L, Low; A, Advanced; M, Middle; DFC, Double First-Class; CE, College English; AEW, Academic English writing; EIAC, English for international academic communication; E, English; ELS, English listening and speaking*.

### Data Collection and Procedure

At the beginning of Week 1, the participants were clearly informed of the purpose and the procedure of the study and consent forms were collected prior to the data collection phase of the study. Therefore, the participants knew clearly that they needed to write about their emotions and the situations in which they experienced these emotions in the online English class, and that the class notes submitted should be taken down during online English learning. For the next 16 weeks, the participants wrote at least one reflective journal every 4 weeks and took class notes. At the end of Week 16, the participants submitted their reflective journals and class notes and the selected participants were interviewed. When conducting interviews, the researcher asked questions by emphasizing the context of online English learning in the spring semester clearly to elicit answers related to online English learning. The timeline of data collection can be seen in Table 3.

**Table 3:** The timeline of the data collection procedure.

**Time**	**Participants' tasks**
Week 1	Be informed of the purpose and the procedure of the study and submit consent forms
	Be clear of the requirement of reflective journals and class notes
Week 1–16	Write at least one reflective journal every 4 weeks and take notes
Week 16	Submit reflective journals and class notes
	Be interviewed

The first primary data source was semi-structured interviews which have been regarded as the most practical way to explore subjective emotional experiences (Ross and Rivers, 2018). The interviews aimed to obtain participants' views on their L2 emotions and the antecedents in an interactive way. The author adopted the WeChat video-calling function in the interview to obtain as much information as that of face-to-face communication. The semi-structured interviews were guided by a list of open-ended questions related to the students' emotional experiences and the antecedents. Before interviewing the six participants, a pilot interview of two EFL learners who are the participants' peers was carried out to verify the outline of the interview questions. The revised outline included four parts: basic information, technical condition of online learning, online class experiences and online situational experiences. Questions in the online situational experiences were optional and served as a supplement of online class experiences when participants found it hard to recall their online learning experiences at the end of the semester. The outline and sample open-ended questions of the interview are presented in Appendix I.

All interviews were carried out in the interviewees' L1, Mandarin Chinese, to avoid language barriers during the interviews. Interviews for each person ranged from 20 to 50 mins, amounting to 3.6 h in total. All interviews were recorded with the participants' permission, which were then transcribed through ifyrec (https://www.iflyrec.com/), a webpage that can transcribe audio recordings into texts (Chiang et al., 2020). The researcher then validated the recordings by listening to the recordings and comparing them with the transcribed texts. Inappropriate transcriptions were revised, and final transcribed text included 30,060 words in total.

The second primary data source is the reflective journal, a way for the participants to rethink their emotional experiences which is especially useful in providing information on the contextual situations of these emotions considering that emotions are aroused by specific situations (Pekrun and Perry, 2014; Sampson, 2019). The participants were required to write a reflective journal at least once a month about the situations that aroused their emotions in the academic setting without any limitation on word number. More specifically, the participants needed to answer two questions. First, what emotions did they experience in their online English class? Second, why did they experience these emotions? At the end of the semester, 40 journal entries with a total of 5,699 words were collected from the six participants.

The supplementary data source was the class notes that the six participants took during the online English classes. This was motivated by the claim that student's class notes can reflect their cognitive engagement which shares interdependence with emotional engagement (Finn and Zimmer, 2012; Philp and Duchesne, 2016). The notes could be taken down according to the participants' willingness without any requirement from the researchers and 15 pages of class notes were finally submitted.

### Data Coding and Analysis

Braun and Clarke's (2006) six-phase-approach for thematic analysis was applied to analyze the data, including familiarization, initial coding, searching for themes, reviewing themes, definition of themes and reporting. QSR International NVivo 12 plus was used for coding and quantitative content analysis by identifying the most codes and references.

Participants' online English learning emotions and antecedents were mainly investigated through semi-structured interviews and reflective journals, both of which were text data sources. Therefore, they shared the same coding scheme and coding procedure. Considering that emotions are aroused in specific situations (Pekrun and Perry, 2014), emotions and the situations in which they occurred were regarded as a whole unit for coding in the initial coding stage (e.g., the quote “*I felt anxious when I couldn't understand”* was chosen as one unit of coding). If the participants mentioned several emotions without explaining the antecedents when answering their emotional experiences (e.g. the quote “*I experienced enjoyment, anxiety and boredom in the online English class*”), this sentence was not regarded as one unit of coding to avoid the overlapping coding of emotions. Since the interviewer would ask follow-up questions about why the participants experienced these kinds of emotions one by one, the specific situation mentioned subsequently by the participant that aroused each kind of emotion (e.g. the quote “*I experienced enjoyment when the points were added”*) was regarded as one unit of coding.

When searching for themes from the data sources, the author coded achievement emotions and the antecedents based on all the units of coding in a bottom-up way. Quotes showing the same emotion and antecedent were coded as one category, and by repeatedly reviewing and comparing these categories, subthemes and themes of emotions and antecedents emerged. For example, “*My nerves tensed up when the teacher called me to answer the question”* was coded as the emotion of anxiety and the antecedent of teaching activities respectively. “*I felt angry when the teacher talked in a monotonous tone”* was coded as the emotion of anger and the antecedent of teaching style. Anxiety and anger were further put into the theme of negative activating emotions, and teaching activities and teaching style were classified as teacher factors belonging to the theme of environmental antecedents. The coding scheme of emotions and their antecedents for the text data sources are presented in the Appendices II and III.

In line with Han and Hyland (2019), the present study also collected supplementary data (i.e. participants' class notes) which could provide information on students' L2 learning contents, activities, cognitive and emotional engagement during online English learning classes. We carefully read the participants' class notes, paid close attention to the contents of the notes and selected those pieces of evidence that could help identify or explain emotions and antecedents. Triangulation could be achieved by referring to different data sources among interviews, reflective journals and class notes (Merriam, 2009).

**Figure 1:**
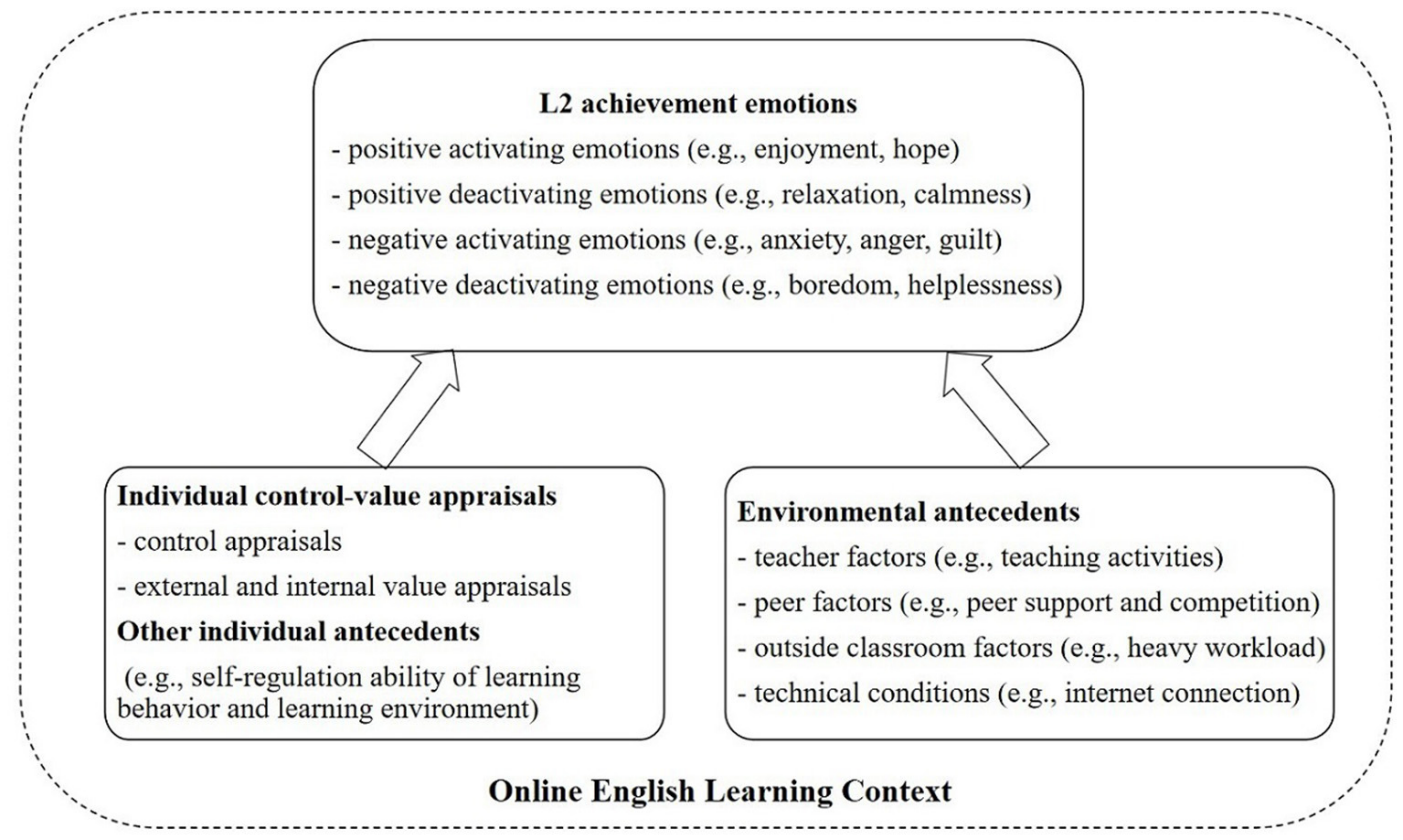
The framework for analyzing the antecedents of L2 achievement emotions in the online English learning context (adapted from Pekrun and Perry, 2014).

After reading and re-reading the data, we developed a framework adapted from Pekrun and Perry (2014) for analyzing the antecedents of online English learning context as shown in Figure 1. Generally, both of the individual and environmental antecedents can lead to online L2 achievement emotions. Individual control-value appraisals play an important role in predicting L2 emotions both in the online language learning environment and the face-to-face context. Other individual antecedents such as the self-regulation ability is of paramount importance considering the online learning context where higher self-regulation ability is needed. As for the environmental antecedents, teacher factors, peer factors, outside classroom factors as well as technical conditions can all affect students' online L2 emotions.

Data reliability was checked by adopting intra-coder reliability based on double coding of the qualitative data during an interval of about 1 month (Li, 2021). The intra-coder reliability was acceptable (*K* = 0.95, *p* < 0.001). The discrepancies of the coding were further analyzed until all coding issues were resolved.

## Results

### Achievement Emotions Experienced in the Online Language Learning Environment

An overall description of the participants' emotions was presented first followed by comparing the six participants to discover similarities and differences. Overall, the 6 participants experienced a total of 15 kinds of emotions involved in all of the four categories of emotional experiences. The achievement emotions mentioned by the six participants in the online English classes based on the interviews and their reflective journals are shown in Table 4.

**Table 4:** Frequency analysis of codes and quotes of the achievement emotions from semi-structured interviews and reflective journals.

**Themes**	**Codes**	**No. of quotes**	**No. of quotes (I)**	**No. of quotes (RJ)**	**No. of participants (*n* = 6) reporting this emotion**
Positive activating emotions	Enjoyment	24	13	11	5
	Hope	10	8	2	3
	Pride	3	2	1	2
	Interest	2	1	1	1
	Curiosity	1	1	0	1
	Gratitude	1	1	0	1
Positive deactivating emotions	Relaxation	20	14	6	5
	Calmness	6	5	1	3
Negative activating emotions	Anxiety	47	29	18	5
	Anger	10	6	4	5
	Guilt	6	4	2	3
	Embarrassment	1	1	0	1
Negative deactivating emotions	Helplessness	7	4	3	4
	Boredom	4	4	0	3
	Disappointment	2	1	1	2
Total	15	144	94	50	6

*I, Interview; RJ, Reflective journal*.

Among the positive activating emotions, enjoyment (24 quotes) and hope (10 quotes) were the most commonly reported emotions. Five participants mentioned that they experienced enjoyment by saying “*I felt happy”* or “*I experienced enjoyment”* and three participants mentioned the words like “*hoped”*, “*expected”*, or “*wanted”* to show their desire to have the English class. For positive deactivating emotions, relaxation (20 quotes) and calmness (6 quotes) were both quite common. For example, both participants B and D said in the interview, “*I felt relaxed after finishing answering the teacher's question”* which showed the emotion of relaxation and participant A said “*I didn't have too much feeling at that time”* which showed her emotion of calmness.

Among negative activating emotions, anxiety (47 quotes), anger (10 quotes) and guilt (6 quotes) were more often experienced than embarrassment (1 quote). All of the six participants except participant E showed their anxiety in various expressions, such as “*I felt worried”* mentioned by participant B, “*I was anxious”*, “*my heart beat faster”* by participant D, “*my nerves tensed up”*, “*I felt nervous”* by participant A. They also mentioned the words such as “*annoyed”*, “*angry”* and “*manic”* to express the emotion of anger. The feeling of guilt was embodied in their expressions such as “*I felt guilty”* mentioned by participants B, D and F. For negative deactivating emotions, the participants experienced helplessness (7 quotes) and boredom (4 quotes) more than disappointment (2 quotes). Four participants mentioned “*I felt helpless”* (Participants A, B, C and F). Boredom was experienced by three participants, among whom participants A and C said “*I felt bored”* and participant B mentioned “*the teaching contents were boring”*.

A closer look at the kinds of emotions and the frequency of quotes could deepen our understanding of individual similarities and differences, which was necessary because the participants selected in the present study were from different educational backgrounds. Table 5 shows the emotions experienced by the six participants and the frequency of quotes.

**Table 5:** Emotions experienced by the six participants and the frequency of quotes.

	**Participant A**	**Participant B**	**Participant C**	**Participant D**	**Participant E**	**Participant F**	**Total**
Enjoyment	2	1	5	9	0	7	24
Hope	0	1	4	0	0	5	10
Pride	1	2	0	0	0	0	3
Interest	0	2	0	0	0	0	2
Curiosity	0	1	0	0	0	0	1
Gratitude	0	1	0	0	0	0	1
Relaxation	5	2	0	6	5	2	20
Calmness	4	1	1	0	0	0	6
Anxiety	10	12	5	14	0	6	47
Anger	2	3	2	1	0	2	10
Guilt	0	1	0	2	0	3	6
Embarrassment	0	1	0	0	0	0	1
Helplessness	2	1	2	0	0	2	7
Boredom	1	2	1	0	0	0	4
Disappointment	0	1	0	0	0	1	2

From Table 5, we can see that most participants experienced at least five kinds of emotions in the online English class except participant E who was pursuing a graduation certificate (i.e., a lower degree compared with the bachelor's degree in China) and only experienced “relaxation” during the whole semester of online English learning. Besides, most participants experienced negative emotions more frequently than positive emotions except the male doctoral participant C and participant E. More specifically, the two doctoral participants C and D and the master participant F experienced enjoyment and anxiety most frequently. The master participant B experienced all of the 15 emotions in her online English learning, with anxiety amounting much higher than the other emotions. The bachelor participant A also experienced anxiety much more frequently than the other emotions.

### Antecedents of the Achievement Emotions

The antecedents of online L2 emotions are not exactly the same in terms of the proportion of individual antecedents and environmental antecedents. The individual antecedents include control-value appraisals and other individual antecedents such as self-regulation which refers to the process of organizing one's actions, thoughts and feelings to achieve personal goals (Usher and Schunk, 2018). As for the environmental antecedents, apart from teacher- and peer-related factors mentioned in the CVT, the present study also found some novel factors that can influence online learning emotions. Overall, the environmental antecedents (98 quotes) accounted for a larger proportion than the individual antecedents of individual control-value appraisals (27 quotes) and other individual antecedents (9 quotes) for all the emotions with exception of hope and guilt. Hope was more related to the individual control-value appraisals in the present study, and guilt concerned more about other individual antecedents such as self-regulation ability. Table 6 shows the antecedents of the most frequently experienced emotions reported by at least half of the participants.

**Table 6:** The antecedents of emotions experienced in the online learning context.

		**Individual antecedents**		**Environmental antecedents**	
		**Individual control-value appraisals**	**Other individual antecedents**	
Positive activating emotions	Enjoyment	2	0	22
	Hope	6	0	4
Positive deactivating emotions	Relaxation	2	0	18
	Calmness	0	0	6
Negative activating emotions	Anxiety	15	3	29
	Anger	1	0	9
	Guilt	0	6	0
Negative deactivating emotions	Helplessness	1	0	6
	Boredom	0	0	4
Total		27	9	98

#### Antecedents of Positive Activating Emotions

The environmental antecedent of teacher factors (18 quotes) was an important source for students' enjoyment, especially when the teacher gave feedback and conducted teaching activities which students liked such as appreciating peers' presentation and self-regulated review. For example, participant C's English class was about academic writing. The teacher's positive feedback on his homework made him feel happy because it was an affirmation of his comprehension and could thus enhance his confidence which would motivate him to write better. In the interview, he mentioned:

…* The teacher would present some students' homework, commend the students for good writing and explain why it is good. I think the teacher's affirmation could boost my confidence, and certainly, I would feel happy*. (Participant C—Interview)

Participant D's English class was English for international academic communication, which covered topics such as chairing an international conference, preparing a presentation, doing a presentation and poster presentation. It was necessary for the students to learn the formulaic language since the purpose of the course was to improve students' academic communication skills in the international context. The teacher taught the students how to communicate in the academic world through the formulaic language and presentation was a common teaching activity for the students to practice what they have learned. Participant D experienced enjoyment when appreciating peers' presentation on chairing an international conference by using the formulaic language they just learned. He wrote in the reflective journal:

…* After the teacher lectured about how to chair an international meeting, she gave us several minutes to prepare for the presentation. Some students were really doing a good job and they were just like chairing a real meeting. I felt happy and sent the emoji of applause into the chat box in Tencent Meeting*. (Participant D—Reflective journal)

**Figure 2:**
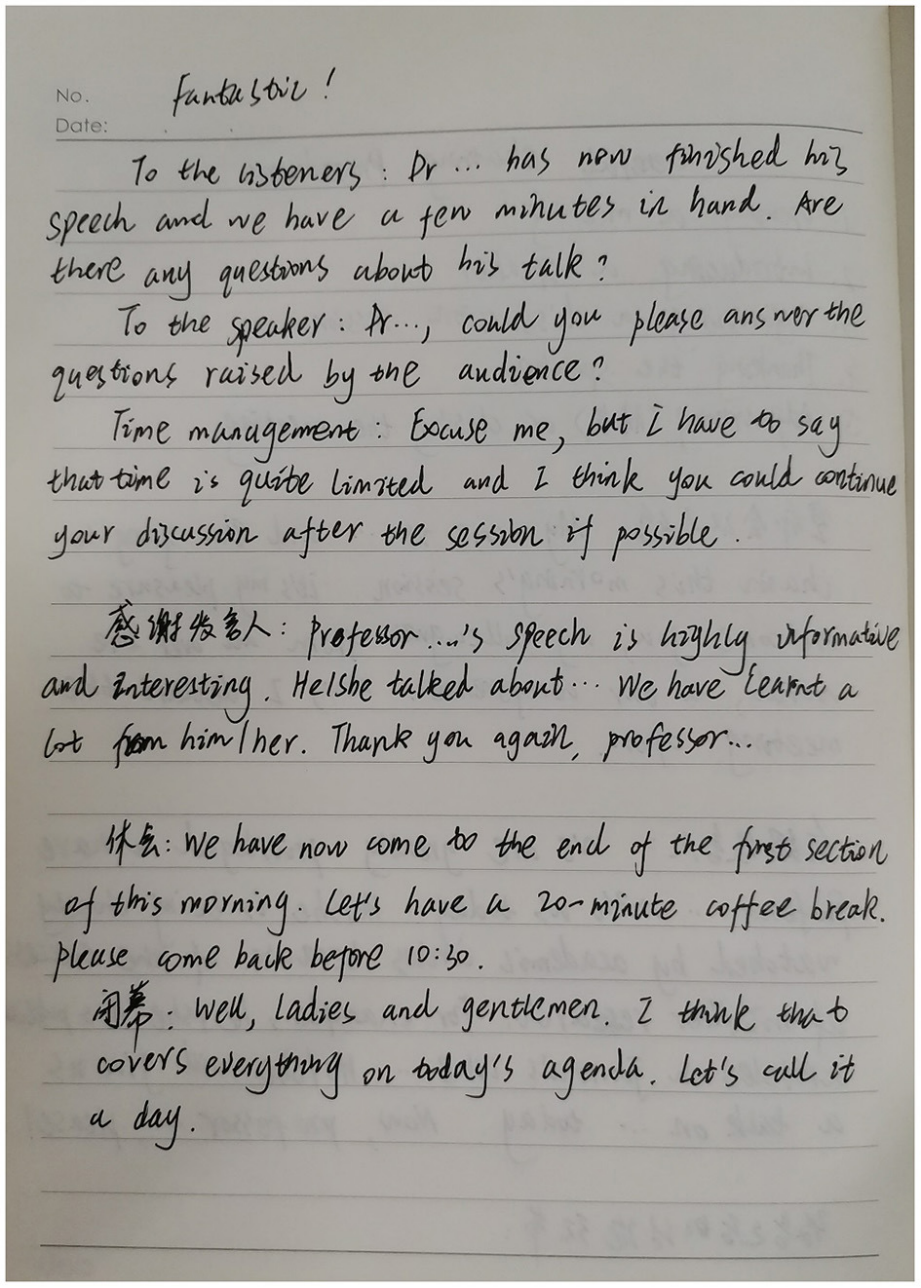
Participant D's class note.

His enjoyment of appreciating classmates' presentation was also reflected in his class note as shown in Figure 2. In his notes, we found not only content-related notes (i.e., notes related to the teaching contents such as the useful phrases when chairing a conference) but also content-unrelated notes (i.e., notes not directly related to the teaching contents such as students' own evaluations of teaching activities) (Participant D—Class note).

When being asked why he wrote a content-unrelated evaluative word “Fantastic” at the top of his class notes in the interview, participant D explained:

…* My classmates could chair an international conference by using the phrases we just learned in the class fluently. I was impressed by their fantastic performance and I enjoyed a lot when listening to their presentations*. (Participant D—Interview)

From participant D's reflective journal and the interview, we could know that the word “Fantastic” in his class notes showed his high evaluation of the classmates' performance. By listening to wonderful presentations, he experienced enjoyment.

Participant B's English class was also about academic English writing. Normally they would read qualified academic research paper and share their opinions and comments on the paper during the class. In the online lessons, self-regulated review, which entails reviewing the academic paper according to one's own pace, brought her enjoyment. She wrote in her reflective journal:

…* Once the teacher said that we could review the paper by ourselves during the class. I was happy that I could learn following my own pace*. (Participant B—Reflective journal)

The environmental antecedent of peer factors (4 quotes) was another source of enjoyment because peer support provided the opportunity of learning from each other. Individual antecedents of value appraisals (2 quotes) also brought enjoyment. Participant A said in the interview:

…* I experienced enjoyment when the points were added*. (Participant A—Interview)

She also wrote in the reflective journal:

…* The teacher would usually check our understanding after her explanation of a language point by sending us a test in the DingTalk platform and we sent the answer in the chat box. The first ten students who answered correctly would get a bonus point. I became happy when I got the point*. (Participant A—Reflective journal)

In participant A's English class, the teacher implemented a different type of bonus measures in the online platform to enhance students' engagement. In the traditional face-to-face classroom, the students could get bonus points if they sat in the first row in the class because students tended not to sit too close to the teacher. In the online class, if the students were among the top 10 who correctly answered a question, they would get bonus points. Participant A experienced enjoyment when she got extra points because the extra points were directly related to the final exam scores and she valued the result of final exam.

Value appraisals (6 quotes) played a vital role in arousing hope as mentioned by students who hoped to have the English class because of its practicality. Participant F mentioned in the reflective journals:

…* I wanted to go abroad in the future which required listening and speaking abilities, so I valued and expected the English class*. (Participant F—Reflective journal)

Her English course was about listening and speaking, involving many common topics that might be encountered in real life such as traveling plans, sports, festivals and so on. Her goal of going abroad and communicating well with others can be achieved by improving her listening and speaking skills in the online English class. She said in the interview:

…* If the topic was important such as traveling, I would look forward to it because I thought I might use these colloquial expressions in the future*. (Participant F—Interview)

Through the two quotes from participant F, we can clearly see that she valued English and the learning activities a lot. Her perceived high external value of learning English (i.e., the practicality of learning English in making her get used to life abroad) motivated her to have the English class and brought her the emotion of hope.

#### Antecedents of Positive Deactivating Emotions

According to Russell (1980), relaxation is the opposite of tension. In the present study, we define relaxation as a positive deactivating emotion experienced without or after a tense situation. The environmental antecedents of the teacher, including teaching method of lecturing (4 quotes), teaching activities of “questions and answers” (3 quotes), and instructional mode of audio conferences (3 quotes) were mentioned more frequently. Participant E experienced only relaxation for the whole semester as she mentioned in her reflective journal:

…* The only emotion I experienced in the class was relaxation*. (Participant E—Reflective journal)

In the interview, when asked why she only felt relaxed during the whole semester, she said:

…* I studied in a college and the English proficiency of the whole class was very low. The teacher knew our proficiency level and didn't want to embarrass us by calling on us to answer questions, so she lectured most of the time without asking questions or interaction*. (Participant E—Interview)

From her explanation we can see that the low proficiency level of the students led the teacher to choose the teaching method of lecturing. No interaction with the teacher in the online classes was the main reason that made her feel relaxed all the time.

Another situation where students could feel relaxed was related to the “questions and answers” session. Participant D mentioned in the interview:

…* After finishing answering the teacher's question, I would feel a little bit relaxed because the teacher would not call me again in a short time*. (Participant D—Interview)

“Questions and answers” was one of the common activities from which the teacher could arouse students' attention and checked their understanding. After finishing answering the question in public, the students can usually feel a sense of relaxation. And the degree of relaxation could be strengthened in the online learning platform when the teacher and students could not see each other.

The instructional mode of audio conferences instead of video conferences in the online learning context could also make the students feel more relaxed, which was reported by participants A, B and F. For example, participant F mentioned in the interview:

…* I liked the instructional mode of audio conferences because we couldn't see each other which made me have more freedom to refresh myself a little bit after a long time period of learning*. (Participant F—Interview)

Compared with the instructional mode of video conferences, which is more like traditional face-to-face learning environment where the teacher and students can see each other, audio conferences leave students with a sense of relaxation and freedom. Without turning videos on, students can listen to the teacher and take notes in the way they like. However, they are lacking in external regulation from the teacher and peers at the same time, which will require more self-regulation ability.

Calmness and relaxation belong to positive deactivating emotions which are close synonyms. According to Barrett and Russell (1998), calmness and relaxation differ in the level of arousal with calmness being a lower level of arousal. Carver and Scheier (2014) further mentioned that calmness is the result of an avoidance approach to a task for fear of failure. In the present study, calmness is defined as a deactivating state resulting from being able to avoid failure in class. Teacher factors (5 quotes) such as teacher characteristics and teaching contents caused calmness in the online English learning in our study. If the teacher was kind, or the teaching contents were easy, students would feel calm in the class since failure to understand or answer the question was not likely to occur in these conditions. Participant C said in the interview:

…* My teacher was kind even when pointing out our problems, so normally I felt calm in the class*. (Participant C—Interview)

#### Antecedents of Negative Activating Emotions

Anxiety mainly came from the environmental antecedents of teaching activities (17 quotes) especially of “questions and answers” as well as technical conditions (4 quotes). “Questions and answers” was regarded as the most challenging situation as mentioned by all of the six participants except participant E. For example, participant B attended the online English classes through WeChat in the form of sending voice messages or text messages, therefore students need several minutes to type or speak about the answer before the teacher finally receives their answers. Participant B was very anxious when thinking about and typing the answer for fear that the teacher would think she was not concentrated if she didn't show her answer in time. She explained in the interview:

…* It was easy for the teacher to misunderstand that you were not online, so you had to either tell her that you were online and thought about an answer or answer the question quickly first and then added supplementary interpretations when the teacher asked the question*. (Participant B—Interview)

In traditional face-to-face classroom, the teacher can see clearly whether the students are absent-minded or not by watching their behaviors. Besides, when the students are answering questions, the teacher will give them enough time to think and express their ideas without making them feel worried, and the teacher can tell whether they can answer the question or not through their facial expressions. Therefore, compared with the face-to-face learning environment, online learning may cause more anxiety under this circumstance.

Besides, technical conditions of the internet connection are necessary for online language learning. With an unstable internet connection, the students may fail to hear the teacher's explanation or answer the teacher's questions on time, leading the teacher to think they are not focused on the lecture during the class. Participant D mentioned in the interview:

…* I felt anxious when there was an internet failure and I was afraid that the teacher would call me to answer the questions at that time*. (Participant D—Interview)

Internet connection is an indispensable guarantee for online learning. When poor internet connection occurs, the students will normally feel anxious especially when the teacher is presenting something important. Waiting for the internet to run well again or changing from the Wi-Fi network to personal hotspot connection takes time, causing the students to be more anxious compared with the face-to-face learning environment where students can hear the teacher clearly without fearing about connection failure.

Anxiety also emerged when students perceived a low control of English (15 quotes). For example, as a doctoral student in engineering, participant C felt anxious and it was difficult for him to understand the lecture because he had a low control in L2 listening, reading and speaking while the teacher spoke English most of the time. Apart from low control of English, individual self-regulation of learning environment (2 quotes) also played a role in arousing anxiety. Participant B saw the importance of independent space in case of the interruption from the family members during the class. She said:

…* You were answering the teacher's question when your mother suddenly brought you something and asked you to eat quickly, which would cause anxiety*. (Participant B—Interview)

Learning environment is of great importance in online learning. However, many students have to take online lessons while their family members are doing other errands. Without an independent and quiet space in the online learning environment, the students may easily be disturbed by others, leading them to have negative emotions.

Anger was mainly aroused by environmental antecedents of the teacher factors (4 quotes), technical conditions (3 quotes) and the outside classroom factors (2 quotes). The teacher's teaching style may cause students' anger by talking “*in a monotonous tone”* (Participant D—Interview) or saying ‘*“well, you know' over and over again”* (Participant B—Reflective journal). Heavy workload outside classroom, for example, “*too many classes a day”* (Participant B—Interview) and “*too much academic research to do”* (Participant C—Interview) could also instigate anger when students were having the online English class.

Students experienced guilt due to a lack of self-regulation (3 quotes) when they failed to concentrate on what the teacher said or the ongoing learning activity. Participants D and F mentioned that they didn't concentrate on the lecture during online English classes because they did something unrelated to the course content. Failing to concentrate on what the teacher said in class made them feel guilty and determined to concentrate more in the next class.

…* Sometimes I was distracted by things around me. Once while the teacher was lecturing, I looked at the pop-up webpage on the computer during the class and then time passed. I didn't follow what the teacher taught and felt guilty*. (Participant D—Reflective journal)

…* Once the teacher asked us to look through a passage in five minutes and raised questions if there were any. I felt guilty when I didn't know the answer of the questions because I checked the phone for five minutes*. (Participant F—Interview).

Online learning is usually taking place *via* computers or smart phones, which can be distractions for the students because of the various functions and applications. Without external regulation from the teacher which students usually have in the face-to-face classroom, online learning requires higher ability in self-regulation. When being attracted by the pop-up webpage on the computer or the messages on the phone and not concentrating on their class content, the students tend to have more negative emotions especially when they find they can't follow the teacher.

#### Antecedents of Negative Deactivating Emotions

The environmental antecedents of peer factors (5 quotes) was the major source of helplessness, especially the lack of peer support. For example, participant A mentioned that there were many pair-work and group-work in the face-to-face English classes, and she was used to discussing with fellow students together and helping each other in class. However, in the online learning environment, it was not common for them to discuss online. No support from the peers in face of difficulties made her feel helpless. And the emotion of helplessness can be intensified when peer competition appeared in the online class, especially when the other students finished a task more quickly. She said in the interview that she felt helpless many times:

…* I felt helpless when no one could tell me how to do it right away like what we used to do during pair-work in the traditional classroom*. (Participant A—Interview)

Compared with face-to-face classroom, one of the disadvantages of online learning environment is that there is less interaction between the teacher and the students and between the fellow students. Lacking in emotional support as well as immediate support related to the learning contents may lead students to feel helpless.

**Figure 3:**
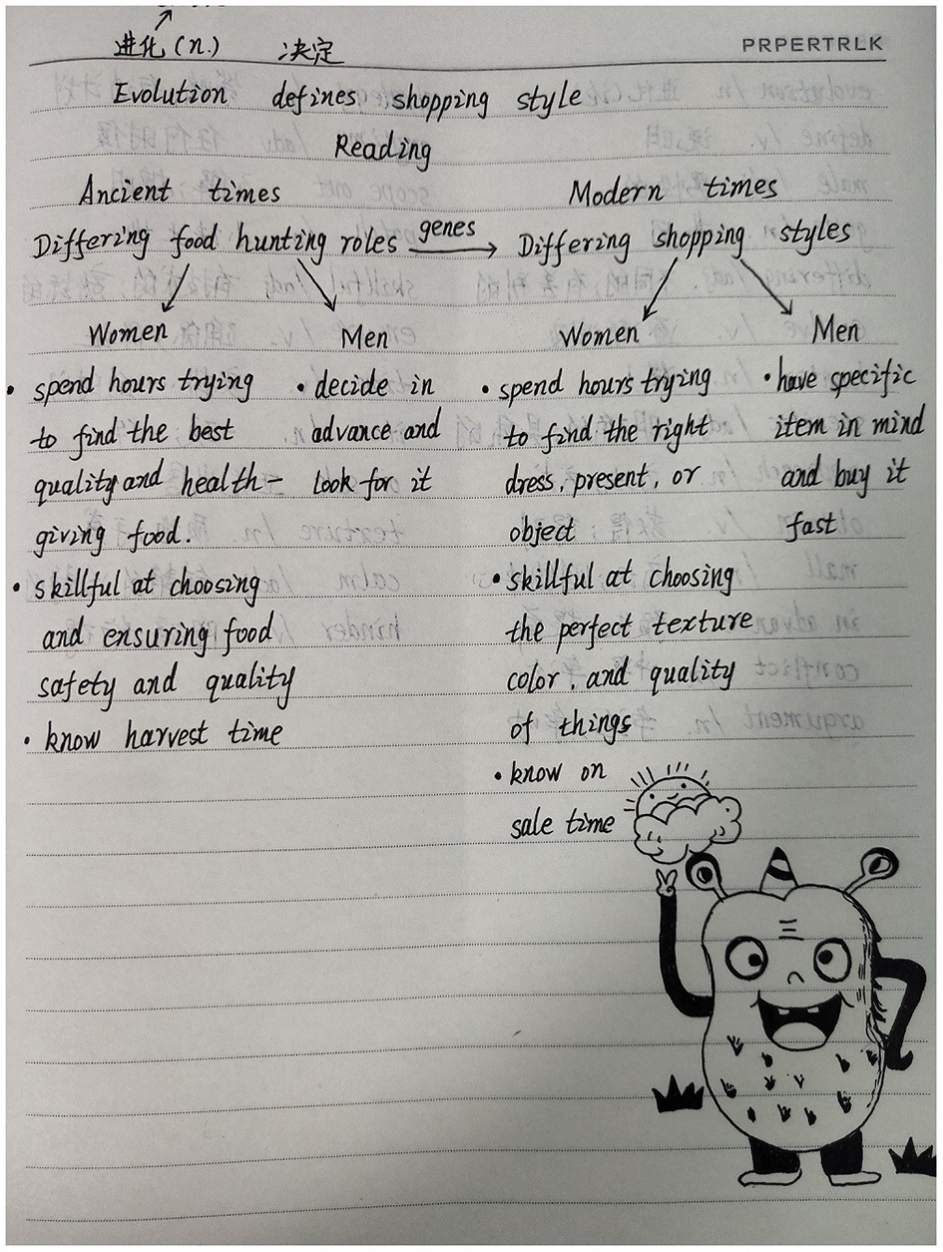
Participant A's class note.

Students mainly attributed their boredom to the teacher (3 quotes), including the teaching contents and teaching style. When the teaching content was boring and had nothing new, students might feel bored. In participant A's class notes, we found some drawings on the pages, such as an animal or a cartoon as shown in Figure 3 (Participant A—Class note).

She explained in the interview:

…* I tried, but I couldn't cheer myself up because the outdated stories in the English book were boring. I just gave up and drew some pictures to kill time. I like drawing*. (Participant A—Interview)

## Discussion

Based on the students' semi-structured interviews, reflective journals and class notes, this study examined the achievement emotions of six Chinese EFL learners in the online English classes and the antecedents of these emotions. Regarding research question 1, the overall finding showed that students experienced a variety of achievement emotions in the online English learning context, which was consistent with findings from Li and Dewaele (2020) and Resnik and Dewaele (2021). As for positive activating emotions, enjoyment was more frequently mentioned than hope, which is consistent with the previous research finding that L2 enjoyment is the most commonly experienced positive emotion (Jin and Zhang, 2018). Half of the participants experienced hope and expressed that they were motivated to have the English class. As MacIntyre and Gregersen (2012) and MacIntyre and Vincze (2017) argue, hope is related to motivation and can facilitate language learning in face-to-face environment. Our results, together with Marchand and Gutierrez (2012), showed that the role of hope in online L2 context was similar to face-to-face environment in that it may motivate students to learn in the class. The positive deactivating emotions of relaxation and calmness were experienced by all the six participants, showing the prevalence of these two emotions.

As for negative activating emotions, anxiety was the most frequently experienced emotion. The present study showed that anxiety was experienced more often than enjoyment. However, Dewaele and MacIntyre (2014) and Jiang and Dewaele (2019) reported the opposite finding. In their studies, students experienced significantly more enjoyment than anxiety in the foreign language classes. The discrepancy might be explained by the special online learning context in our study, which can trigger more anxiety due to the poor internet connection and students' perceived low level of interaction and support. On the one hand, unstable connection of the internet may cause negative emotions. According to Coman et al. (2020), 69.4% of the participants experienced technical problems such as connection difficulties, signal loss and blurred sound, all of which could cause negative emotions (e.g. anxiety) when the students failed to follow the teacher and perceived a low control of the teaching contents (Daniels and Stupnisky, 2012). On the other hand, in the online learning context, there was less interaction between the teacher and the students or between fellow students compared with face-to-face learning environment (Russell, 2020). As a result, students' perceived low level of interaction and support from peers and teachers could make them feel isolated and anxious. Anger ranked as the fourth frequently mentioned emotion in the present study, similar to Resnik and Dewaele's (2020) study which ranked anger as the fifth frequently occurring emotion after fun, boredom, happiness and love. Despite the similar ranking, the antecedents of anger were different considering the online learning environment. Anger was mostly related to poor internet connection in our study while it was mainly attributed to the teacher factor in Resnik and Dewaele (2020). Guilt was experienced by three students in our study and feeling guilty motivated them to listen carefully and behave better next class. Teimouri (2018) also found that feeling guilty can motivate L2 students to accept responsibility instead of blaming others, take corrective actions, and make more efforts to learn.

As for negative deactivating emotions, half of the participants mentioned that they experienced boredom in the online learning environment. L2 boredom has also been frequently experienced in the face-to-face learning environment with its negative effect on motivation and engagement in the learning activities (Li, 2021). Helplessness was experienced by the students in our study largely due to a lack of peer support especially in the online learning environment. Feeling helplessness for many times may lead to learned helplessness, which will cause negative effects such as frustration and demobilization on the students (Raufelder et al., 2017).

Many kinds of emotions such as enjoyment, hope, anxiety and boredom occur in both the face-to-face instructional environment and the online instructional environment (Li and Dewaele, 2020; Resnik and Dewaele, 2021). However, the online instructional environment poses special challenges to the students such as lower levels of sense of achievement and higher boredom (Li and Dewaele, 2020) and requires higher self-regulation ability (Li and Zhou, 2020). As a result, what differentiates emotions experienced in the face-to-face instructional environment and the online instructional environment is the frequency and the intensity of these emotions rather than the type of emotions. For example, negative emotions (e.g., boredom) can be exacerbated in the online learning environment when there is no physical presence of the teacher and more distractions from the environment (Stodel et al., 2006; Han and Gao, 2020) and positive emotions such as enjoyment were lowered in the online learning environment compared with the face-to-face instructional environment (Resnik and Dewaele, 2021).

Regarding research question 2, it was found that apart from control-value appraisals, self-regulation was also an indispensable part of individual antecedents. Besides, the environmental antecedents not only included teacher and peer factors but also technical conditions and workload outside classroom. In general, the environmental antecedents took up a higher percentage in leading to L2 achievement emotions in the online learning environment than the individual antecedents.

As for the positive activating emotion of enjoyment, environmental antecedents played a more important role than individual antecedents in our study. More specifically, teacher factors accounted for a large part in the environmental antecedents. This result is consistent with Dewaele et al. (2017) and Jiang and Dewaele (2019) which demonstrated that foreign language enjoyment was predicted more by teacher-related variables than the learner-internal variables. Apart from the teacher-related variables such as friendliness of teacher, teacher's joking, and teacher's frequent use of foreign language reported in Dewaele et al. (2017) and Jiang and Dewaele (2019), the present study showed that teacher feedback and interesting teaching activities were also important sources of enjoyment, confirming Jiang (2020) which also revealed that effective teacher feedback and interesting teaching activities such as ice-breaking activities in the first English class and content-related games could arouse students' enjoyment, and supporting the CVT which claimed that feedback and motivational quality (e.g., interesting teaching activities can motivate the students to participate and learn) are important environmental factors influencing emotions (Pekrun and Perry, 2014). For the positive activating emotion of hope, perceived value is the major antecedent. In the present study, the perceived external value of learning English for writing qualified SCI papers and going abroad led to hope. According to the CVT (Pekrun, 2006), students who had a high control or valued academic studying generally tended to have positive emotions.

As for positive deactivating emotions, the effect of environmental antecedents was more obvious than the individual antecedents. The favorable learning environment created by the teacher through instructional mode of audio conferences instead of video conferences, the teaching methods of lecturing, appropriate level of difficulty of the teaching contents and kindness of the teacher can make the students feel relaxed and calm in the classroom in our study. This result echoed previous findings that teacher friendliness can arouse students' positive emotions and teacher-centered lessons contribute to reducing negative emotions (Dewaele et al., 2019b; Jacob et al., 2019). Furthermore, after a tense situation such as answering questions, students may experience relaxation or calmness. According to Pekrun et al. (2007), emotions can influence cognitive resources (e.g., attention allocation, working memory, planning). Negative emotions can lead to a narrowed attentional focus (Fredrickson, 2001). Viau-Quesnel et al. (2019) also revealed that negative emotions can exert an adverse effect on reasoning because negative emotions detract cognitive resources which are necessary for reasoning. However, positive emotions, on the other hand, can broaden the scope of attention and cognition, enhancing emotional well-being (Fredrickson, 2001). Positive deactivating emotions, though with a lower level of arousal, can renew or refresh students' emotional well-being and cognitive resources, which may have positive effects on learning and achievement outcome (Brackett and Rivers, 2014; Goetz and Hall, 2014).

As for negative activating emotions, our study showed that individual control-value appraisals exerted a significant influence on anxiety. More specifically, perceived low control may bring about negative emotions such as anxiety in the present study, showing that perceived control independently can bring about emotions, and improving students' perceived control of the learning material is necessary for decreasing negative emotions (Pekrun, 2006).

Self-regulation, another individual antecedent, resulted in negative emotions including guilt and anxiety in our study, complementing the CVT in two ways. On the one hand, the CVT mainly claimed the reciprocal relationship between positive emotions and self-regulation while related negative emotions to external regulation from teachers or peers (Pekrun et al., 2007). Our study showed that there existed an interaction between negative emotions and self-regulation as well. Students with lower ability of self-regulation would be distracted easily and then perceive a lower control of what was taught, inducing negative emotions such as guilt, which would in turn motivate them to regulate themselves better next time. Enhancing students' self-regulation of their learning behavior is helpful for reducing their negative emotions especially in the online learning environment where there is not much external regulation to rely on. On the other hand, the present study showed that studying in an interrupted place was important for efficient learning in the online context, confirming that environment structuring (e.g., choosing an appropriate place to learn English online to avoid distraction) is a necessary dimension of self-regulation in the online English learning (Zheng et al., 2016).

Compared with individual antecedents mentioned above, environmental antecedents played a more important role in arousing anxiety in the present study. This was inconsistent with Jiang and Dewaele (2019) which reported that foreign language anxiety is predicted more strongly by learner-internal variables than teacher-related variables. This difference may be explained by the variation in conditions of the online learning context and the traditional face-to-face learning context (Artino and Jones, 2012; Daniels and Stupnisky, 2012). Weak internet connection can easily arouse negative activating emotions such as anxiety and anger.

In addition to the technical condition of internet connection, other environmental antecedents of negative activating emotions include teacher factors, peer factors and workload outside classroom. The “questions and answers” session deserves special attention since our study showed that answering questions in class instigated anxiety the most, which was consistent with previous findings in EFL context in China and America, confirming that being singled out to answer questions can cause anxiety (Horwitz et al., 1986; Liu, 2006). Besides, our study supported the CVT in that the environmental antecedents such as cognitive quality (i.e., the structure, clarity and difficulty of teaching contents) and goal structures can arouse achievement emotions (Pekrun, 2006). In the present study, students experienced anxiety due to the low cognitive quality and competitive goals among peers, both of which caused them to feel a lack of control of the teaching contents. In addition, outside classroom factors also need to be considered. In line with Russell (2020) which emphasized anxiety aroused by the outside classroom factors of the COVID-19 pandemic and students' home situation, this study also found that the heavy workload outside classroom can arouse negative activating emotions such as anger, complementing the CVT which mainly focused on the inside classroom antecedents.

As for negative deactivating emotions, the environmental antecedents, especially teacher factors and peer factors, are responsible for boredom and helplessness respectively. When the motivational quality of the contents is poor or the teacher repeatedly talks in a monotonous way, students are prone to feel bored. Similar results were reported by Pawlak et al. (2020), which showed that repetitiveness and monotony can result in boredom, and involving more interesting topics and activities can offset boredom. In addition, our study also found that insufficient peer support can lead to helplessness. Failing to get appropriate support on time may induce negative emotions such as helplessness especially in the online language learning environment which requires additional teacher and peer support (Russell, 2020).

## Conclusion

The present study was conducted in the semester when all the students in China took online language courses because of the lockdown situation during the COVID-19 pandemic. We explored the achievement emotions of six Chinese EFL learners in the online English classes and the antecedents of these emotions. Anxiety was experienced most frequently by the participants, followed by enjoyment, relaxation, anger, hope, helplessness, calmness, guilt and boredom in order. Overall, positive emotions can be caused by individual value appraisals and environmental antecedents of teacher factors such as positive feedback, interesting teaching activities and instructional mode of audio conferences. Negative emotions are related to individual control appraisals and low online self-regulation ability as well as environmental antecedents such as “questions and answers” session, lack of peer support and poor internet connection.

While Pekrun (2006) developed the CVT mainly from the traditional face-to-face environment, the present study applied this theory to the online learning environment. Our study supports the CVT in that individual control-value appraisals exert an influence on achievement emotions, and environmental antecedents can arouse emotions as well. We also extend the CVT by including other specific factors that could play a role in arousing emotions in the online context. First, the technical condition of internet connection is of paramount importance in the online environment to ensure effective teaching and learning. Secondly, environmental antecedents such as workload outside classroom can also influence students' emotions in the English class. Thirdly, students' self-regulation of learning behavior and learning environment are critical factors for successful learning in the online learning context. The analytical framework of the antecedents of achievement emotions in the online language learning context can be applied to investigate online L2 emotions for future studies.

The pedagogical implications of the study are two folds. First, in order to promote positive activating emotions, teachers can cultivate the perceived value, especially the intrinsic value of English learning by motivating students through engaging contents. In addition, positive feedback is necessary to enhance positive emotions such as enjoyment and hope. In order to decrease negative emotions, it is advisable for the teachers to improve students' perceived control over the learning materials by designing tasks with high cognitive quality and providing appropriate teacher and peer support. In this way, students can follow the learning materials at a steady pace and increase learning efficiency. Besides, a supportive environment for students is needed to enable them to concentrate on the contents (e.g., decreasing chances of answering questions in public or peer competition). Moreover, it is necessary to teach the students how to self-regulate their learning during the classes to achieve desirable learning outcome, especially in the online learning context.

Several limitations of the present study should be taken into account. Firstly, although we collected data from different sources of semi-structured interviews, reflective journals and class notes to triangulate the results, the number of participants, though acceptable, was rather small (Patton, 1990; Dörnyei, 2007; Mackey and Gass, 2016). Secondly, the data sources in the present study were all collected from the perspective of the participants and relied heavily on the their recall of past experiences. A combination of data collected from the researchers' and teachers' perspectives such as online class observation and the teachers' lesson plan can make the qualitative study more solid. Thirdly, the interviews were carried out only once at the end of the semester, which may not reflect different kinds of emotions at different periods during the semester. Future studies could be conducted with a longitudinal design to investigate students' emotions at different time periods. Finally, future studies could also explore the dynamics of emotions through an idiodynamic method to examine the moment-to-moment fluctuations of emotions (Gregersen et al., 2014; Elahi Shirvan and Talebzadeh, 2017).

## Data Availability Statement

The raw data supporting the conclusions of this article will be made available by the authors, without undue reservation.

## Ethics Statement

Ethical review and approval was not required for the study on human participants in accordance with the local legislation and institutional requirements. The patients/participants provided their written informed consent to participate in this study.

## Author Contributions

YY and ZG discussed the idea and design of the study. ZG collected the data and drafted the manuscript. YY and YH together revised the draft of the manuscript. All authors contributed to the article and approved the submitted version.

## Funding

This study was supported by the Ministry of Education in China Project of Humanities and Social Sciences Research under Grant Number 20YJA740052 and the Fundamental Research Funds for the Central Universities in UIBE (76211260) awarded to YY.

## Conflict of Interest

The authors declare that the research was conducted in the absence of any commercial or financial relationships that could be construed as a potential conflict of interest.

## Publisher's Note

All claims expressed in this article are solely those of the authors and do not necessarily represent those of their affiliated organizations, or those of the publisher, the editors and the reviewers. Any product that may be evaluated in this article, or claim that may be made by its manufacturer, is not guaranteed or endorsed by the publisher.

## Footnotes

^1^“Double First-Class” universities refer to the universities listed in the “Double First-Class” project initiated by the Chinese Ministry of Education, including 42 world-class universities and 95 universities which have their own first-class disciplines in China (http://www.moe.gov.cn/jyb_xwfb/s5147/201709/t20170922_315006.html).
