# Supplementary material for: Exploring Chinese EFL Learners' Achievement Emotions and Their Antecedents in an Online English Learning Environment
Source: Front Psychol. 2021 Oct 15;12:722622. doi: 10.3389/fpsyg.2021.722622 (PMC8554019; doi:10.3389/fpsyg.2021.722622)
Supplement: Supplementary file 1 [file Table_1.pdf]

APPENDIX I

Semi-structured interview guide

Part 1): Basic information

What is the English course you are taking this semester?

Does your teacher mainly speak in English, in Chinese or in a bilingual way?

How do you rate your English proficiency, low, middle or high level?

Part 2): Technical condition of online learning

How do you have the online English class, in the way of video, audio or online texts? Which way do you prefer and why?

Have you or your teacher experienced any problems in using online teaching platforms? How do you feel about them?

Part 3): Online class experiences

How does your English teacher design the class?

What are the major activities in your English class?

If you are asked to describe your emotional experiences in an English class this semester, which words will you use to describe them?

Which factors do you think lead you to have this emotional experience?

Have you ever experienced the emotion of …?

Part 4): Online situational experiences (optional)

How do you feel when you are called to answer questions?

What is your emotional experience when the teacher responds to your answers?

What is your emotional experience when doing a presentation?

How do you feel when doing listening/reading comprehension in the online English learning class?

What is your emotional experience during self-regulated review?

How do you feel when there is an internet breakdown?

APPENDIX II

Coding scheme for the various emotions in interviews and reflective journals

**Theme**	**Coded for**	**Selected quotes**
Positive activating emotions	Enjoyment	*"I experienced enjoyment when the points were added."* Interview-Participant A
	Hope	*"I wanted to go abroad in the future, so I valued and expected the English class."* Reflective Journal-Participant F
Positive deactivating emotions	Relaxation	*"I felt relaxed after finishing answering the teacher's question."* Interview-Participant D
	Calmness	*"My teacher was kind even when pointing out our problems, so normally I felt calm in the class."* Interview-Participant C
Negative activating emotions	Anxiety	*"My nerves tensed up when the teacher called me to answer the question."* Reflective Journal-Participant A
	Anger	*"I felt annoyed when the internet connection was poor."* Reflective Journal-Participant C
	Guilt	*"I felt guilty when I didn't know the answer of the questions because I checked my phone for five minutes."* Interview-Participant F
Negative deactivating emotions	Helplessness	*"I felt helpless when no one could tell me how to do it right away like what we used to do during pair-work in the traditional classroom."* Interview-Participant A
	Boredom	*"I felt bored and didn't want to do homework because there was too much academic research to do."* Interview-Participant C

APPENDIX III

Coding scheme for the antecedents of various emotions in interviews and reflective journals

Subtheme	Coded for	Selected quotes
**Theme: environmental antecedents**		
Teacher factors	Teaching activities	*"My heart beat faster when the teacher called my name to answer questions."* Reflective Journal-Participant D
	Teaching contents	*"The class cannot be interesting all the time, after all, the teaching contents were boring."* Interview-Participant B
	Teaching style	*"I felt angry when the teacher talked in a monotonous tone."* Interview-Participant D
	Teaching methods	*"The teacher lectured during the whole class sometimes, making me feel relaxed."* Reflective Journal-Participant D
	Instructional mode	*"We couldn't see each other through audio conferences. I felt more relaxed this way than face-to-face teaching."* Interview-Participant A
	Teacher feedback	*"I think the teacher's affirmation could boost my confidence, and certainly, I would feel happy."* Interview-Participant C
	Teacher characteristics	*"My teacher was kind even when pointing out our problems, so normally I felt calm in the class."* Interview-Participant C
Peer factors	Peer support	*"I experienced enjoyment because some students learned well and were willing to help me during the cooperation."* Interview-Participant C
	Peer competition	*"I felt anxious when other groups had finished, but our group hadn't reached an agreement."* Reflective Journal-Participant B
Outside classroom factors	Heavy workload	*"I felt bored and didn't want to do homework because there was too much academic research to do."* Interview-Participant C
Technical conditions	Internet connection	*"It might be the breakdown of your own network while the teacher or other students' internet run well. This drove me manic."* Interview-Participant A
**Theme: individual antecedents**		
Control appraisals	Listening ability	*"I felt worried because it was difficult for me to have the English-only academic writing class, and sometimes I couldn't understand."* Interview-Participant C
	Speaking ability	*"I wanted to say the word 'review', but I couldn't think of it when answering the question. I felt anxious at that time."* Reflective Journal-Participant F
Value appraisals	External value appraisals	*"I experienced enjoyment when the points were added."* Interview-Participant A
Self-regulation	Self-regulation of learning behavior	*"I looked at the pop-up webpage on the computer during the class and then time passed. I didn't follow what the teacher taught and felt guilty."* Reflective Journal-Participant D
	Self-regulation of learning environment	*"You were answering the teacher's question when your mother suddenly brought you something and asked you to eat quickly, which would cause anxiety."* Interview-Participant B
